# Distribution of Hepatitis C Virus Genotypes Among Patients With Hepatitis C Virus Infection in Hormozgan, Iran

**DOI:** 10.5812/hepatmon.14324

**Published:** 2013-12-16

**Authors:** Seyedeh Farzaneh Mousavi, Seyed Hamid Moosavy, Seyed Moayed Alavian, Hajar Eghbali, Hamidreza Mahboobi

**Affiliations:** 1 Shahid Mohammadi Hospital of Hormozgan, University of Medical Sciences, Bandar Abbas, IR Iran; 2 Baqiyatallah Research Center for Gastroenterology and Liver Diseases, Baqiyatallah University of Medical Sciences, Tehran, IR Iran; 3 Middle East Liver Diseases Center (MELD Center), Tehran, IR Iran; 4 Infectious and Tropical Diseases Research Center, Hormozgan University of Medical Sciences, Bandar Abbas, IR Iran; 5 Department of Phychology, Payame Noor University, Tehran, IR Iran

**Keywords:** Hepatitis C, Genotype, Iran

## Abstract

**Background:**

More than 170 million people in the world are infected with Hepatitis C virus (HCV). Determination of HCV genotype before starting the treatment is required, because HCV genotype affects the course of treatment and drug dosage

**Objectives:**

We aimed to evaluate HCV genotypes among patients with positive results for anti-HCV in Bandar Abbas from 2011 to 2012.

**Patients and Methods:**

Five hundred and nine consecutive patients with established chronic HCV infection referred to Behavioral Diseases Consultation Center, Blood Transfusion and Center for Special Diseases from March 2011 to March 2012 were enrolled in this cross sectional study. Five mL of peripheral blood was taken from precipitants and viral RNA extracted after plasma separation. Hepatitis C virus RNA was detected by reverse transcriptase-nested polymerase chain reaction (RT-nested PCR) assay and then HCV genotypes analyzed using restriction fragment length polymorphism (RFLP) method.

**Results:**

In overall, 509 patients enrolled to this study. The mean age of these patients was 38.87 ± 9.55 years ranging from 1 to 90 years. Routs of transmission were: 238 (46.7%) inject of substance, 149 (29.3%) unknown rout, 62 (12.2%) blood transfusion, 50 (9.8%) sexual contact, and 10 (2%) mother to child. Frequency of HCV genotypes were: 316 (62.1%) 1a, 117 (23%) 1b, and 76 (14.9%) 3a. there was no significant association between HCV genotypes and gender, educational degree, risk factor of Hepatitis C, job, monthly income, HIV infection, Hepatitis B virus (HBV) infection, Intravenous drug injection, and underlying disease (P > 0.05).

**Conclusions:**

This results the same as many similar studies demonstrated that common HCV genotypes in Iranian patients were 1a, 3a and 1b, respectively. Patients with 1a and 1b genotypes have lower responses to interferon treatment, and it is reasonable to perform early screening to diagnose and determine HCV genotype for effective treatment and diagnose high-risk cases.

## 1. Background

More than 170 million people worldwide are infected with Hepatitis C Virus (1). Hepatitis C is a blood-borne disease and the main risk factor is exposure to infected blood or blood products such as: transfusion with unscreened blood and blood products, needle-sharing among Intravenous (IV) drug abusers, and needle-stick injuries in health care workers. The most common cause of transmission is needle or syringe sharing among drug abusers with a prevalence between 31 to 89 percent according to different geographical areas. Risk of infection for some groups of patients such as, patients on hemodialysis, those with hemophilia infants of mothers with HCV infection, and multipartner individuals are dramatically higher. Prevalence of HCV among patients on hemodialysis in Iran is 5.5% to 55.9% in different cities, although this number for patients with hemophilia is 15.65 to 76.7%. 

HCV comprises six major genotypes (genotypes 1 to 6) and several subtypes (a, b, c, etc.) which have different geographical distribution (2). It is needed to determine HCV genotype prior to treatment, because this is the genotype which determines treatment period and drug dosage. In addition, genotype is an important preventive agent for probability of viral tolerance, and probability of obtaining and sustained virologic response (3).

There exists a certain geographical distribution of HCV genotypes. Genotype 1 is the most common one in the US and Europe; and genotypes 2 and 3 have the lowest prevalence in these regions and genotypes 4, 5, and 6 are rare. Genotype 3 is the most common in India, Far East and Australia. Genotype 4 is the most common in Africa, the Middle East, and it seems that the most common European type is related to IV drug abusers and homosexual males. Genotype 5 is the most common in southern Africa, and genotype 6 is the most common in Hong Kong, Vietnam and Australia (4-7). It sounds that the prevalence and incidence of different HCV genotypes in geographical regions and periods of time are due to distribution mode and evolution of risk factors (8).

Several studies were performed in Iran to explore this geographical distribution. In a study conducted by Joukar et al. (2009), in Guilan it was found that the most common genotypes among patients on hemodialysis are 1a and 3a (59.38%) (9). Data gathered from 16 provinces in a study by Hajia et al. (2009), in Tehran indicated that the most prevalent genotype was 3a (46.6%) and then genotype 1 (1a, 1b with the prevalence of 25.73% and 17.47%) ranked the second. The findings of this study during four years showed that HCV infection with genotypes 1b, 3a, has increased from 12.2% and 38.9% to 18.9% and 46.5% in the fourth year (10). Another study performed by Samimi-Rad et al. (2008) in Markazi province revealed that the overall prevalence of Hepatitis C was 5.4% and the prevalent subtype among patients on hemodialysis was 1a (50%), then genotypes such as 4 (25%), 3a (12.5%) and 1 b (12.5%) ranked second to fourth (11). Currently this disease is treated by pegylated IFN-α and Ribavirin which has severe side effects and its outcome depends totally on the HCV genotype (12-14).

## 2. Objectives

Based on the fact that HCV genotype varies in different geographical regions and since the type of effective treatment and response to treatment varies in different genotypes of this virus, these authors decided to investigate the distribution of HCV genotypes of patients with positive results for HCV in Hormozgan, 2011 to take more effective treatment decisions in this field.

## 3. Patients and Methods

### 3.1. Study Population

In this cross sectional study 509 patients with positive results for anti-HCV antibody and HCV-RNA tests referred to Behavioral Diseases Consultation Center, Blood Transfusion and Center for Special Diseases were enrolled between March 2011 and March 2012. The present study was approved by committee of medical student thesis of Hormozgan University of Medical Sciences (HUMS). Patients were informed about the study and then an informed consent was obtained from each participant. Inclusion criteria were positive results for plasma genomic HCV-RNA and anti-HCV antibodies. For all patients a two-part check list was prepared as follows: 1. Questions regarding demographic information, 2. Questions about route of infection, time of diagnosis, presence of Blood born coinfections, underlying disease, addiction to drugs or previous abuse.

### 3.2. Sample Collection and RNA Extraction 

About 5 milliliter of peripheral blood was taken from each participant into EDTA-containing vacutainer tubes. Plasma was obtained from whole blood by centrifugation and storage at -80 °C for later detection. Viral RNA was extracted from plasma sample using High Pure Viral Nucleic Acid Kit (Roche Diagnostics GmbH, Mannheim, Germany), according to the manufacturer’s instructions. 

### 3.3. cDNA Synthesis and Hepatitis C Virus Genotyping Using Restriction Fragment Length Polymorphism Assay

The Genomic HCV-RNA in plasma sample was detected using reverse transcriptase-nested polymerase chain reaction (RT-nested PCR) method. cDNA synthesis from viral RNA and two round nested-PCR were performed as previously described elsewhere (15). 

Hepatitis C virus-genotypes were analyzed in RT-nested PCR products using restriction fragment length polymorphism (RFLP) assay. The nested-PCR and RFLP assay were performed using primers from the 5´-untranslated region (5´-UTR) as described previously by Pohjanpelto et al. in details (16). The second round of PCR products (173-bp) with digested PCR products by special restriction enzymes and appropriate controls with 50bp molecular weight size marker (Fermentas GmbH, St. Leon-Rot, Germany) were visualized using 3% agarose gel electrophoresis. Different HCV Genotypes were determined based on the molecular weight of each fragment of PCR product. The results of HCV genotyping by RFLP assay were confirmed by sequencing of 5´-UTR of HCV. The 5´-UTR was amplified from viral RNA extracted from plasma specimens of 10 randomly selected participants with Pfu DNA polymerase and the PCR products were sequenced using dye termination method by ABI 3730 XL sequencer.

### 3.4 Statistical Analysis

Data was analyzed by descriptive statistical methods (frequency, Standard Deviation, Mean) and statistical test Chi-square using SPSS version 13. 

## 4. Results

Totally 509 subjects took part in the present study. The average age was 38.87 ± 9.55 years ranging from 1 to 90 years. Disease transmission methods in this study were IV drug abuse (%56.7, N = 238), unknown (29.3%, N = 149), transfusion (12.2%, N = 62), sexual contact with infected individuals (9.8%, N = 50), and mother to child (2%, N = 10) which this vertical transmission is more than previous results from other parts of Iran. Prevalence of HCV genotypes in these subjects was as follows: 316 (62.1%) 1a, 117 (23%) 1b, and 76 (14.9%) 3a. Distribution of genotypes of hepatitis C with sex, HIV infection, hepatitis B (HBV) infection and underlying diseases has been illustrated in Table 1. 

**Table 1.** Presentation of 509 Iranian Patients Regarding Their HCV-Genotype

HCV-Genotype	Presentation Patients			P value
	3a (N = 76)	1b (N = 117)	1a (N = 316)	
**Male/Female, (%)**	70/6 (92.1)	112/5 (95.7)	296/20 (93.7)	Non-significant
**Transmission of HCV**				Non-significant
Post-transfusion	14 (18.4)	14 (12)	34 (10.8)	
IV drug abuse	34 (44.7)	55 (47)	149 (47.2)	
Sexual	7 (9.2)	15 (12.8)	28 (8.9)	
unknown	19 (25)	32 (27.4)	98 (31)	
Prenatal	2 (2.6)	1 (0.9)	7 (2.2)	
**Underlying Disease**				Non-significant
Renal failure	0 (0.0)	0 (0.0)	1 (0.3)	
HBV Coinfection	1 (1.3)	3 (2.6)	21 (6.6)	
HIV Coinfection	13 (17.1)	25 (21.4)	65 (20.6)	
**Complications**				Non-significant
Cirrhosis	0 (0.0)	0 (0.0)	1 (0.3)	

Also distribution of a variety of Hepatitis C genotypes with level of education, job has been shown in Table 2. Our findings indicated that there was not any significant association between various hepatitis C genotypes and gender, level of education, risk factors for hepatitis C, job, HIV infection (103 patients), HBV infection (25 patients), and the presence of underlying diseases (P > 0.05). 

**Table 2.** Presentation of 509 Iranian Patients Regarding Their HCV-Genotype and Relationship to Demographic Data

Presentation Patients	HCV-genotype, No. (%)			P value
	1a (N = 316)	1b (N = 117)	3a (N = 76)	
**Occupation**				Non-Significant
Unemployed	108 (34.2)	41 (35)	25 (32.9)	
Employee	108 (34.2)	39 (33.3)	28 (36.8)	
Free job	44 (13.9)	21 (17.9)	11 (14.5)	
Housekeeper	5 (1.6)	3 (2.6)	2 (2.6)	
Student	9 (2.8)	3 (2.6)	1 (1.3)	
Scholar	7 (2.2)	3 (2.6)	3 (3.9)	
Laborer	34 (10.8)	6 (5.1)	3 (3.9)	
Retired	1 (0.3)	1 (0.9)	3 (3.9)	
**Education**				Non-Significant
Illiterate	92 (29.1)	34 (29.1)	16 (21.1)	
Lower than diploma	61 (19.3)	25 (21.4)	16 (21.1)	
Diploma	92 (29.1)	36 (30.8)	25 (32.9)	
Licentiate	70 (22.2)	22 (18.8)	19 (25)	
Higher than licentiate	1 (0.3)	0 (0.0)	0 (0.0)	

## 5. Discussion

The most frequent genotypes in our study were 1a (62.1%), 1b (23%), and 3a (14.9%) respectively.

Some studies in Iran have reported different results. Genotype 3a (61.2%) was reported as the most frequent genotype in Isfahan province followed by 1a (29.5%) and 1b (5.1%) genotypes (17). Alavian et al. reported the distribution of HCV genotypes as follows; genotype 1 in 57%, genotype 3 in 35%, and genotype 2 in 1%, and mixed genotype in 4% of Iranian patients with thalassemia (18).

The information about the prevalence of different HCV genotypes in Iran is variable according to the study population and geographical setting. We have summarized the results of some studies in Table 3. 

As shown in Table 3 the most frequent HCV genotypes in Iran were 1a and 3a. As shown in this table the frequency of HCV genotypes is variable based on study population and geographical areas. Some studies have focused on patients on hemodialysis and those with hemophilia, while others investigated patients with HCV referred to clinics. 

**Table 3.** HCV Genotypes Frequency in Iran

Study	Population	Province	No. of Patients Studied	Results
**Assarehzadegan et al. (2009) (19)**	Patients on hemodialysis	Khuzestan province	34	1a (41.1%), 3a (35.2%), 1b (23.5%)
**Samimi Rad et al. (2007) (20) **	Patients with thalassemia and those with hemophilia	Markazi province	2 patients with thalassemia and 23 with hemophilia	Hemophilia: Genotype one in 50%, three in 18.2%, two in 4.54%, and mixed in 27.3%, Thalassemia: two (40%) had positive results for HCV RNA and one sample was subtype 3a.
**Keyvani et al. (2007) (21) **	Patients with hepatitis C referred to hepatitis clinics	Tehran province	2231	1a, (39.7%), 3a (27.5%), and 1b (12.1%)
**Kabir et al. (2006) (22)**	Patients with HCV	Tehran	156	1a (37.8), 3a (28.9%), 1b (16.7%).
**Hosseini-Moghaddam et al. (2006) (23)**	Patients on hemodialysis	Tehran	66	3a (30.3), 1a (28.8%), 1b (18.2%), 4 (16.7%)

Also the results of studies in our neighborhood countries are different. One study in Turkey have reported genotype 1b in 84.7% of patients with HCV followed by 3a (4.2%) and 1 (3.8%), which is not compatible with our results (24). In Pakistan the most frequent genotypes were 3a (40.96%), 3b (15.66%), and 1a (9.63%) respectively (25). In Saudi Arabia genotype 4 (50%) was the most common genotype followed by genotype 1b (40.9%), and 1a (9.1%) (26, 27). In China, one study on patients with HCV and HIV coinfection reported genotype 6a(39.3%) as the most frequent genotype followed by genotypes 1b (24.7%), 3b (18%), and 3a (9.8%) (28).

The present findings showed that there was no significant association between a variety of hepatitis C genotypes and gender, level of education, and risk factors for hepatitis C, job, income, HIV infection, HBV infection, IV drug abuse and presence of underlying diseases. In addition, the present findings indicated that there was no significant association between the types of HCV genotype and the underlying diseases. The results of the study by Amini et al. and Kabir et al. are in agreement with the present findings. They found no significant difference between the HCV genotypes and risk factors and demographic characteristics (22, 29). Keyvani et al. indicated an increase in frequency of genotype 1b with increasing age (21).

We did not find any significant association between HCV genotype and underlying diseases or complications of the disease. It is shown that patients with genotypes 1a and 1b tended to have more severe liver disease and lower response to interferon therapy (30). Also patients with genotype 1b are at higher risks for hepatocellular carcinoma (31). Alavian's study on patients with thalassemia and simultaneously HCV infection, showed that dominant genotype 1 may result in splenectomy and increased ferritin in blood (18). 

The importance of determination of the most frequent genotypes in each area is the differences in treatment response and prognosis of patients (32-36).

Our study showed that the most frequent genotypes of hepatitis C in Bandar Abbas are 1a, 1b, and 3a respectively. This frequency is different in other provinces in Iran and the neighboring countries. Therefore, we recommend more studies on the treatment options available for patients in each area based on the most frequent genotypes.
